# The Complement System in the Central Nervous System: From Neurodevelopment to Neurodegeneration

**DOI:** 10.3390/biom12020337

**Published:** 2022-02-21

**Authors:** Ying Chen, John Man Tak Chu, Raymond Chuen Chung Chang, Gordon Tin Chun Wong

**Affiliations:** 1 Department of Anaesthesiology, LKS Faculty of Medicine, The University of Hong Kong, Hong Kong SAR, China; aprilcy@connect.hku.hk (Y.C.); jmtchu@hku.hk (J.M.T.C.); 2 Laboratory of Neurodegenerative Diseases, School of Biomedical Sciences, LKS Faculty of Medicine, The University of Hong Kong, Hong Kong SAR, China; 3 State Key Laboratory of Brain and Cognitive Sciences, The University of Hong Kong, Hong Kong SAR, China

**Keywords:** complement, astrocytes, microglia, neurons, neurodevelopment, neurodegeneration, neuroinflammation

## Abstract

The functions of the complement system to both innate and adaptive immunity through opsonization, cell lysis, and inflammatory activities are well known. In contrast, the role of complement in the central nervous system (CNS) which extends beyond immunity, is only beginning to be recognized as important to neurodevelopment and neurodegeneration. In addition to protecting the brain against invasive pathogens, appropriate activation of the complement system is pivotal to the maintenance of normal brain function. Moreover, overactivation or dysregulation may cause synaptic dysfunction and promote excessive pro-inflammatory responses. Recent studies have provided insights into the various responses of complement components in different neurological diseases and the regulatory mechanisms involved in their pathophysiology, as well as a glimpse into targeting complement factors as a potential therapeutic modality. However, there remain significant knowledge gaps in the relationship between the complement system and different brain disorders. This review summarizes recent key findings regarding the role of different components of the complement system in health and pathology of the CNS and discusses the therapeutic potential of anti-complement strategies for the treatment of neurodegenerative conditions.

## 1. Introduction

The complement system is foundational to the innate immune response in defending the body against invading pathogens by phagocytosis or by the activation of the adaptive immune system. In the CNS, however, the complement system protects the brain from not only pathogens but other potentially harmful stimuli such as aberrant proteins and cellular debris [1]. Findings from studies presented in this review will show that complement components are produced by both neurons and glial cells. This local production of complement factors may be a developmental advantage as it enables a more rapid response than reliance on peripheral production and diffusion through the blood–brain barrier (BBB). Under normal circumstances, the activation of the complement system in the CNS consists of over 30 complement factors under tight regulation [2]. However, when this well-tuned regulatory machinery malfunctions, aberrant complement factors can exacerbate neurological symptoms of brain conditions and accelerate the development of aging-related or neurodegenerative diseases [3,4,5]. Emerging evidence suggests that higher levels of complement factors are present in developing and degenerating brains and perform novel functions in neurodevelopment and contribute to the pathophysiology of neurodegenerative diseases [3,6,7,8]. Therefore, an understanding of endogenous complement production and regulation in the brain can provide insights into aberrant neurodevelopment and the genesis of neurodegeneration (Table 1). This review will therefore provide a summary of major aspects of the activation and regulation of complement pathways, followed by a more focused discussion of the role of complement in the CNS and in neurogenerative conditions.

## 2. Complement Component and Pathways

**Figure 1:**
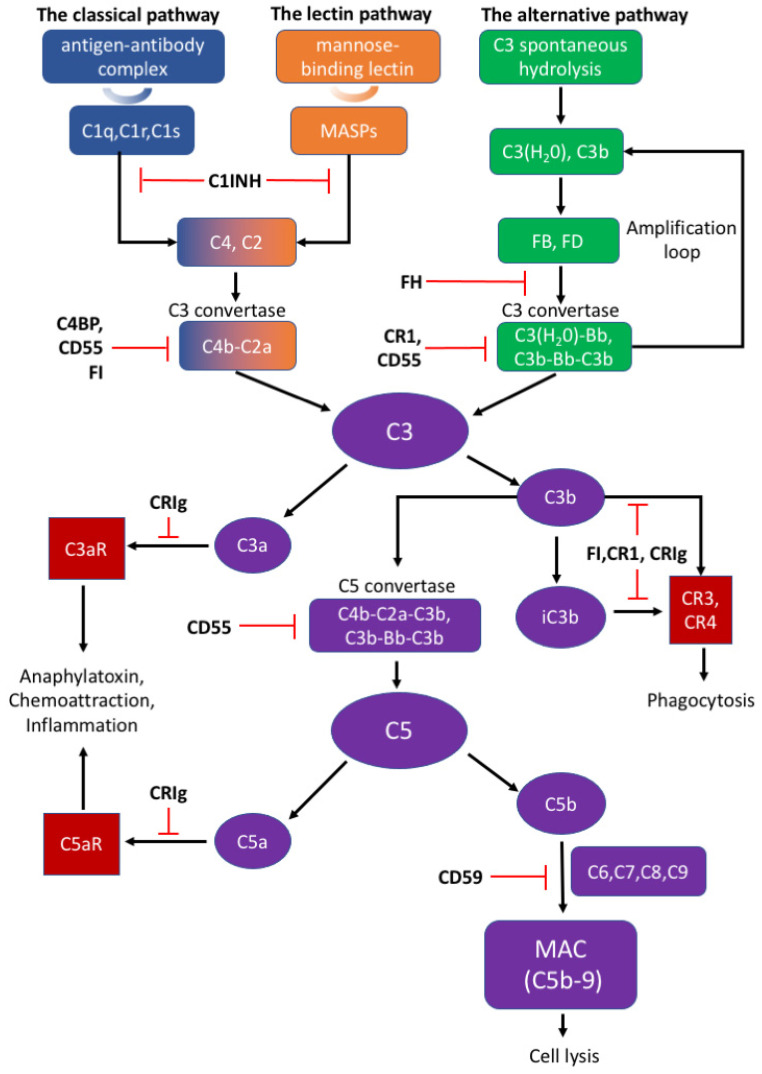
The activation and regulation of the complement system.

There are three distinct pathways that activate the complement system: the classical, lectin, and alternative pathways (Figure 1). The classical pathway is initiated by C1q complex formation when C1q recognizes antigen–antibody complex [78]. The lectin pathway is analogous to the classical pathway, which begins from the binding of mannose residues by mannose-binding lectin (MBL). C1q complex and mannose-binding lectin associated proteases (MASPs) cleave C2 and C4 into C2a and C4b, followed by C3 convertase (C4b–C2a) formation [79,80]. The alternative pathway can be initiated from C3 hydrolysis or from C3b directly. Once factor B binds with C3(H_2_O) or C3b, it is cleaved by factor D to become C3(H_2_O)Bb or C3b-Bb as another C3 convertase, which further produces more C3b to amplify the complement cascade [81]. Thus, the alternative pathway accounts for around 80–90% of complement activation [9,10,12,13]. C3 convertases cleave C3 into C3a and C3b; C3a can induce downstream activation by binding to C3aR, and C3b binds to CR3 or CR4 to mediate phagocytosis [82]. C3b can further combine with C4b–C2a and C3b-Bb to generate C5 convertases (C4b–C2a–C3b and C3b–Bb–C3b) and activate the terminal pathway which is regarded as the effector pathway of the complement system [6,10,11,13]. Similar to C3, C5 convertases cleave C5 into C5a and C5b; C5a is another important chemotactic protein similar to C3a, and C5b assembles with C6–9 to generate the membrane attack complex (MAC) [79,80].

The appropriate activation of the complement cascades rely on a series of tightly regulated soluble and membrane-bound complement inhibitors. Such complement inhibitors have been shown to successfully control complement activation in animal models with CNS disorders [4]. Soluble complement inhibitors include C1 inhibitor (C1INH), complement inhibitor C4b binding protein (C4BP), factor H (FH) and factor I (FI) while complement receptor type 1 (CR1), CRIg, CD55, and CD59 belong to the membrane-bound complement inhibitors [83]. C1INH, also known as SERPING1, inactivates the proteolytic effects of the C1 complex, MASP1, and MASP2, to inhibit the classical and lectin pathways [79,84]. Deficiency of C1INH can affect normal neurodevelopment, indicating that appropriate complement system function is important to the CNS [1]. C4BP can bind to C4b and act as a decay-accelerating factor [83]. Moreover, FH is the dominant regulatory factor of the alternative pathway and by competitively binding to C3b, it destabilizes C3 and C5 convertase both on cell surfaces and in plasma [79]. FI can permanently inactivate C3b and iC3b and degrade C3 convertase by cleaving the C4b component aside by cofactors such as FH, C1INH, and C4BP [79]. CR1 is a single-pass membrane glycoprotein expressed on different cells and acts as a cofactor to FI in accelerating the dissociation of C3 convertase [79,81]. Rodents bear only the complement receptor type-1 related protein (Crry) in place of CR1 [85]. Another potent inhibitory factor CRIg converts C3a or C5a into inactive forms, which impairs signal transmission through the C3a or C5a receptors [79]. An inactive C3b product (iC3b) also interacts with CR1 or CRIg for further degradation [78,79]. CD55 is widely expressed on various cells and inhibits the formation of and accelerates the dissociation of C3 convertases among all three complement pathways [78,83]. Different from other complement inhibitors, CD59 binds to C5b-8 on the host cell surface and blocks the binding and polymerization of C9 to prevent MAC formation [79].

It is well-known that 90% of soluble serum complement components are derived from the liver constitutively and activated immune cells are an important source of inducible complement protein [81]. However, the CNS may not be exposed to the same composition of complement components as in the periphery due to selective restriction of the BBB [1,2,17]. Recent studies reveal that complement components could be synthesized locally by resident cells in the CNS that provide “immunosurveillance” to maintain normal functionality in the brain [86].

### 2.1. Complement Production in the CNS

Astrocytes, as the most abundant glial cell in the brain, are the main source of complement components in the CNS. Levi-Strauss and Mallet were the first to show that astrocytes are capable of producing C3 and factor B using primary rodent astrocyte cultures [22]. Gasque et al. used human astrocyte cell lines to further demonstrate that the expression of C3, C4, FH, FB, C1INH, and the terminal pathway components in the presence of TNF-α or IL-1β, and especially IFN-γ [23,24,50]. Similarly, the RNA-seq of astrocytes found in brains affected by Alzheimer’s disease (AD) and normal aging reveals microglial-derived IL-1α, C1q and TNF-α induced C3 upregulation in astrocytes, which is regarded as the biomarker of neurotoxic reactive astrocytes [25,87,88,89]. Astrocytes also synthesize regulatory proteins, such as CD55, CD59, FH, FI, C4BP, and membrane cofactor protein (MCP), which render astrocytes more resistant to the cytolytic effects of complement [8,66]. As for complement receptors, astrocytes are able to express anaphylatoxin receptors C3aR and C5aR, but not CR3 or CR4 [8,28,29].

While C3 is mainly derived from astrocytes, C1q contains microglia as its primary source, produced when stimulated by IFN-γ or IL-1β [2,90]. Microglia can also express complement receptors CR1, CR3, CR4, C3aR, and C5aR, but not CR2 [8,20,31]. In the CNS, microglia are the only source of CR3 and CR4, and when interacting with C3b/iC3b or C4b, respectively, promotes microglial phagocytosis [8,24,91]. C3–CR3 mediated phagocytosis plays a vital role in neurodevelopment and in neurodegenerative diseases [92,93]. In addition, C3aR and C5aR are both anaphylatoxin receptors and play a key role in chemotaxis and mediation of inflammatory responses [66,93]. Moreover, the activation of C3aR and C5aR contributes to tau and amyloid pathology in AD, synapse loss and cognitive dysfunction [35,54,63,69,93,94,95]. In addition, microglia were shown to produce C3 and FH [2,74,75].

Conventionally, the neuron is regarded as a victim of complement activation. However, neuronal cells also produce complement components [2]. Data from neuronal cell cultures, the mouse brain and the postmortem brains of AD patients have shown that neurons can express complement components such as C1q, C2, C3, C4, C5, C6, C7, C8, bearing in mind that C1q is essential to neurodevelopment [9,10,11,26,90]. In the murine encephalitis model, neuronal expression of complement components, including C3aR and C5aR, is upregulated and associated with synaptic stripping and motor impairment [96]. Neuronal C3aR and C5aR expressed during different neurodevelopmental stages exert different functions to facilitate brain maturation [65]. Moreover, neurons synthesize a series of regulatory proteins, such as C1INH, CD55 and CD59, which protect neurons from complement attack [2].

### 2.2. The Complement Regulatory Mechanisms in the CNS

As the importance of the complement system in the CNS becomes more apparent, an increasing amount of work has been performed to characterize the expression of complement components in the brain. Here, we summarize the recent findings of complement transcriptional regulatory mechanisms under various conditions, which will enhance our understanding of the complement system in CNS health and therapeutic implications for neurological conditions.

NF-κB is a pro-inflammatory transcriptional factor that mediates a series of cytokines in the CNS. A recent study showed that astrocytic NF-κB mediates C3 upregulation in the AD brain [31]. Another study revealed that the RAGE–p38 MAPK–NF-κB axis is responsible for the higher levels of astrocytic C3 in diabetic mouse models, while in the HIV infected mouse brain, NF-κB-dependent IL-6 is necessary for C3 promoter activation [97,98]. Furthermore, FH is the downstream target of NF-κB in microglia and is downregulated by miRNA155, 125, and 146. Interestingly, these miRNAs are also under the transcriptional control of NF-κB [74,75]. CCAAT/enhancer-binding protein β (CEBPβ) is another transcription factor mediating C3 expression in neural cells from the hippocampal region [99,100]. TNFα induces C3 upregulation via NF-κB and CEBPβ, which is counter-regulated by higher histone hyperacetylation [101,102]. Moreover, the circadian clock gene Baml1 modulates astrocytic C3 and neuronal C4b expression via the BMAL1-REV-ERBα axis in the brain [103]. However, astrocytic transcriptional factor Nrf-2 suppresses C1q and C4 expression to protect against brain damage and cognitive impairment in a cerebral hypoperfusion mouse model [104]. In addition, TGF-β is a key regulator of neuronal C1q expression in the developmental brain, which is essential for maintaining normal synaptic pruning and the visual system function [12]. While in the CNS of HBV-infected mice, IFN-γ/arginase 1 signaling mediates microglia M1 polarization and C1q expression [105]. Interestingly, a novel complement factor sushi domain-containing protein 4 (Susd4) has been found with abundant expression in neurons. In addition to blocking neuronal C1q expression, Susd4 also binds to C1q and C1q complex with sushi motifs to impede C3 convertase [106]. Another sushi domain protein SRPX2 also functions as an endogenous neuronal complement inhibitor, which protects neurons against synapse elimination during neurodevelopment [107].

## 3. Complement System in the Developing Brain

Neurodevelopment is a complicated process involving various signaling pathways and molecular mechanisms. It has been shown that complement components are upregulated during pre- and post-natal developmental stages of the CNS, while dramatically decreasing in the matured brain. This development-dependent dynamic change is essential for appropriate development, while imbalances in the complement cascades may result in vulnerability to developmental diseases, such as schizophrenia and autism [1,2,6,7,85].

### 3.1. The Complement System in Normal Neurodevelopment

The functions of the complement system in the developmental brain are increasingly being elucidated and generally fall into three categories: progenitor proliferation, neuronal migration, and synaptic pruning [7]. Complement components can promote the proliferation of progenitor or stem cells in different organs including the CNS [108,109]. Based on the high expression of C5a in the CSF and C5aR in the neocortex, the C5a–C5aR pathway promotes embryonic neural proliferation via the atypical PKCς pathway, while inhibition of this C5a–C5aR interaction causes microstructural abnormalities and behavioral changes [51]. In comparison, C3aR shares an analogical evolutionary relationship and expression manner with C5aR, but pharmacological blockade of C3aR promotes neural proliferation [27]. Although grossly normal neurogenesis is present in C3aR deficient mice, memory recall is nevertheless impaired, indicating a mechanism different to that of C3aR is necessary for normal cognition [27]. These results suggest that both C3aR and C5aR contribute to the homeostasis of neural progenitor proliferation.

The complement system plays a critical role in neuronal migration which when impaired, is associated with developmental diseases such as schizophrenia. The knockout or knockdown of MASP1, MASP2 or C3 results in deficient neuronal migration and abnormal cortical thickness, which is reversible via C3 cleaved products, indicating these complement molecules mediate neuronal migration [28]. Intriguingly, the inhibitory complement proteins C1INH and SERPING I contribute to radial migration and neural proliferation in the prenatal stage. The same study also indicated that C3aR and C5aR pathways are involved in neuronal migration [52].

Considering that brain size, neuronal number and synaptic connections increase sharply during postnatal development, minimizing inappropriate neuronal connections is important to neuronal wiring and plasticity. Synaptic pruning is one of the methods to eliminate synapses that are “too weak” or “too strong” [1,2,7,85]. Complement components, especially C1q, are crucial to synaptic refinement. Punctate C1q immunoactivity is localized to synapses in the developing brain, while genetic deficiency of C1q or C3 suppresses synaptic pruning [9]. The change in neuronal C1q occurs in a TGF-β dependent manner [12]. Nevertheless, emerging evidence suggests that synaptic elimination is more likely to rely on microglia-mediated engulfment through the C3/CR3 pathway and follows an activity-dependent fashion [61]. Similar results are also seen in spinal motor circuits [62].

### 3.2. The Complement System in Neurodevelopmental Conditions

#### 3.2.1. Schizophrenia

Schizophrenia is a heritable psychiatric disorder, characterized by a range of symptoms including delusion, hallucinations and disordered thinking. This disease manifests typically in adolescence or early adulthood, an important and sensitive period of neurodevelopment, resulting from both genetic vulnerability and environmental factors. Considering the reduction of synapse connectivity, it has been speculated in recent decades that the complement system is involved in the pathogenesis of schizophrenia [82,85]. According to the genome-wide association study (GWAS), complement components C4, Sushi Multiple Domains 1(CSMD1), and C2 were identified as genetic markers of schizophrenia risk [47]. C4A and C4B, two isotypes of the human gene, are both upregulated in schizophrenia, with C4A expression higher than C4B. Additionally, each C4 allele can exhibit a distinct level of schizophrenia risk regardless of which haplotype it appears in, thus providing support that the C4 gene is related to schizophrenia risks [46,82]. Subsequently, an increase in C4 expression promotes neuronal complement deposition and microglia-mediated synaptic engulfment [110]. This excessive synaptic pruning impairs synaptic connectivity [110,111]. To illustrate the significance of these findings and further investigate how C4A affects neural circuits in vivo, another study generated a mouse line expressing human C4A isoforms, which shows a reduced synaptic density, increased microglial engulfment, and altered behavior compared to WT mice [112]. In addition to the genetic aspects, environmental factors, such as prenatal inflammation or viral infections, can also result in the exposure to activated complement cascades, impairment of synapses and cognitive symptoms that resemble schizophrenia [82,113].

#### 3.2.2. Autism or Autism Spectrum Disorder

Autism or autism spectrum disorder (ASD) is a neurodevelopmental disorder marked by poor social interaction, communication deficits and repetitive behaviors. ASD is a complex and multifactorial condition influenced by environmental, genetic, and immunological factors. However, the etiology or the pathology of ASD remains largely unknown, and animal models or laboratory tests are also lacking. This knowledge gap encouraged the conduct of serum proteomic studies to identify altered proteins or biomarkers present in autistic patients [114,115]. Initially, the serum protein profile of children with autism revealed different expression of apolipoprotein B 100 (ApoB100), complement factor H related protein 1 (FHR1), fibronectin 1 (FN1), and C1q [15]. The following studies also observed that various complement components increased in patients with ASD, including C1q C3, C5 and C8 [29,114,116]. Another study showed that C3-related microglial phagocytosis plays a vital role in sex-specific behavior formation, while aberrations of C3 will affect neurodevelopment and social behavior in adolescence [30]. All these data indicate that dysregulation of complement cascades may be involved in the pathogenetic mechanism or could represent a biomarker for ASD [117].

## 4. The Complement System in Neurodegenerative Diseases

The pattern of complement expression changes throughout life, with high levels appearing in young or aged brains, but dramatically decreases and remains relatively low and stable in healthy adult brains [63]. In addition to the complement system’s function and contribution during neurodevelopment, increased levels of complement components are also observed in neurodegenerative diseases, including AD, multiple sclerosis (MS), amyotrophic lateral sclerosis (ALS), Parkinson’s disease (PD), Huntington’s disease (HD), and perioperative neurodegenerative disorders (PNDs). Emerging evidence suggests that the activation of complement system plays a critical role in the pathological mechanisms of these neurodegenerative diseases. Thus, in this section, we discuss some key findings of various complement components in the progress of these neurodegenerative diseases.

### 4.1. Alzheimer’s Disease

AD is the most common neurodegenerative disease characterized by progressive cognitive dysfunction and memory loss [118]. The characteristic AD pathological changes comprise β-amyloid (Aβ) plaques, tau aggregation, neuroinflammation and synaptic losses [119]. The loss of synapses is regarded as an early pathological event and correlates with cognitive decline resulting from reactive microglial-dependent synaptic engulfment.

In the AD brain, the immunoactivity of C1q is localized within neurons in the frontal cortex and the hippocampus and is associated with Aβ plagues, which suggests that upregulation of C1 is activated by Aβ plaques [120,121]. However, a recent study applied spatial transcriptomics and in situ sequencing to analyze the genomic changes and cellular characteristics within the plague-surrounding area, displaying the upregulation of the classical pathway molecules with the presence of reactive astrocytes, disease-associated microglia, and oligodendrocytes [122]. C1q deficiency in APP mice reduces glial cell activation and attenuates the decrease of synaptophysin seen in APP mice at 12–16 months of age when Aβ plaques are present [13]. Similarly, an unbiased quantitative proteomic study revealed that the C1q protein level significantly increased in tau-enriched synapses, which further contributes to the increased microglial engulfment of synapses and the reduced synapse density [14]. Moreover, anti-C1q antibodies can suppress these changes in cultured neuron and tau-P301S mice [14]. These observations suggest that C1q contributes to the development of neuropathological changes in AD via microglia-dependent synaptic elimination.

The central complement molecule C3 additionally plays a vital role in the development of AD pathology. Analogous to C1q, an increase in C3 localized to synaptic puncta in AD mice is also observed [32]. Furthermore, an elevated C3 level is detectable in the brain and CSF of AD patients, closely correlating with amyloidosis and tauopathy [32,33,36]. Although previous studies showed that elevated C3 is induced in astrocytes and neurons afflicted with AD pathology, accumulating evidence reveals that the increased C3 is mainly derived from astrocytes. The transcriptome profile of sorted astrocytes shows a noticeable elevation of C3, along with other classical complement components [36]. Furthermore, the analysis of astrocyte-derived exosomes from AD patients displays high C3 protein levels, along with other complement components [34]. This change in C3 levels in AD mouse models may follow the activation of the NF-κB pathway in astrocytes [31]. Another study revealed the relationship of C3 and reactive astrocyte phenotypes, indicating that the elevated C3 can act as a biomarker for neurotoxic reactive astrocytes in AD and other neurodegenerative diseases [25]. In the presence of abnormal Aβ plaques or tau pathology, microglial CR3 recognizes C3 and its cleaved forms C3b/iC3b and initiates microglial phagocytosis that results in synaptic loss, which can be impeded by either neutralization or genetical deletion of the C3–CR3 pathway [32,33,36]. Apart from CR3 signaling, the anaphylatoxin receptor C3aR also contributes to the pathogenesis and cognitive deficits in AD. Unlike CR3 which is only expressed on microglia, C3aR can be produced by neurons, astrocytes as well as microglia and mediates pro-inflammatory responses and chemotaxis [93]. When C3 interacts with neuronal C3aR, it causes neuronal and dendritic morphology disruptions and synaptic plasticity aberrations [31]. When C3 binds to microglial C3aR, it alters expression of immune networks, mediates neuroinflammation, and even modulates amyloid and tau pathology in AD mice [35,68].

Another anaphylatoxin C5a and its receptor C5aR are additionally involved in AD pathogenesis and cognitive impairment. In the AD brain, the increased C5aR immunoreactivity colocalizes with neurofibrillary tangles (NFTs), hyperphosphorylated tau, and dystrophic neurites, indicating that C5aR signaling may be related to tau pathology [123]. Furthermore, the transcription profile of sorted microglia from AD mice reveals that genetic deletion of C5aR reverses the inflammatory polarization of microglia and significantly suppresses phagocytosis [53]. Notably, long-term oral administration of a C5aR antagonist significantly reduces hyperphosphorylated tau and NFTs, amyloid deposits, and surrounding activated glial cells [54]. Moreover, an active C5a-target vaccine triggers endogenous production of anti-C5a antibodies in AD mice and efficiently improves memory function via suppressing neuroinflammation and amyloidosis [55]. This indicates the C5a–C5aR pathway acts as a pro-inflammatory mediator in AD pathology.

Intriguingly, C3 activation is beneficial to Aβ clearance. The C3–CR3 signaling pathway accelerates fibrillar Aβ clearance by microglial phagocytosis [37]. Moreover, the lack of C3 facilitates Aβ plaque deposition by reducing phagocytic microglia [124]. Similarly, overexpression of an endogenous anti-C3 inhibitor protein Crry promotes Aβ plaque formation [73]. Moreover, a clinical study reveals that relatively lower C3 and FH levels in the CSF are regarded as prognostic biomarkers to predict faster cognitive decline in the mild cognitive impairment (MCI) stage of AD [76]. Thus, C3 plays a dual role in AD pathology and further investigations are required to explain these differential effects of activation and modulation [125].

### 4.2. Multiple Sclerosis

MS is one of the most prominent autoimmune demyelinating diseases in the CNS, affecting multiple systems and presenting with various symptoms including progressive cognitive impairment and even dementia [126]. Although the underlying pathogenesis remains yet to be fully understood, the pathological changes of MS are characterized by inflammatory responses and demyelination [5,127]. The immune and inflammatory responses in the MS brain are complex and involve peripheral cell infiltration, BBB disruption, resident glial cell activation working together. Evidence pointing to the possibility of increased complement factors being involved in pathogenesis of MS continue to emerge [126,127,128,129,130]. Therefore, only the role of complement components in demyelination and neurodegenerative symptoms in this section will be discussed.

Multiple complement components are found in the brain of MS, such as C1q, C3, C3b/iC3b, MAC, C1NH, C3aR, and C5aR which is consistently present within MS plaques, with C1q and C3 signals predominantly restricted to reactive astrocytes [16]. The RNA sequencing profile indicates that the complement cascade, especially C3, is significantly upregulated in astrocytes adjacent to the optical nerve in EAE and correlates with the severity of neuronal and axonal loss [40]. Another study showed that C1q is increased in neurons, C3b/iC3b mainly increased in neurons and glial cells, and C3aR and C5aR in microglia, all of which surround MS lesions [18]. Additionally, higher levels of complement components are observed in patients’ serum and CSF [131,132]. Given the presence of BBB disruption and leukocyte infiltration into brains affected with MS, peripheral complement components may also contribute to damage in the CNS [126].

Whereas previous research focused more on axonal and neuronal damage, attention has now shifted towards synaptic changes in MS. A previous study observed that the increase of C1q in neurons and deposition of C3 in synapses are surrounded by phagocytotic microglia, which negatively correlates with the level of synaptic markers [17]. Knock-out of C3, but not C1q, dramatically attenuates loss of synapse and microglial activation in the EAE model [39]. Similarly, another study shows that even when C1q and C3 are both upregulated in MS and EAE brains, C3 is predominately localized on synaptic terminals, and overexpression of the anti-C3 inhibitory protein Crry attenuates loss of synapses from microglial engulfment [38]. This suggests that synaptic elimination by microglia in MS relies on the alternative pathway, which may be directly activated by C3 binding to myelin [126]. Interestingly, in the basolateral amygdala (BLA), one of the main centers for emotional control, microglia activation and complement cascades act in an opposing way and lead to increased synaptic activity through less synaptic pruning [133]. As for the terminal complement pathway, C5a–C5aR signaling and MAC are present in higher levels in MS brain tissue and also contribute to EAE progression [60,72]. Nevertheless, the inhibition of MAC formation impedes disease progression more effectively than blocking C5aR [60]. Thus, strategies targeting complement factors, especially C3 and MAC, bear potential value as therapeutic agents.

### 4.3. Amyotrophic Lateral Sclerosis

ALS, also known as motor neuron disease, is a progressive neurodegenerative disease without efficient treatment at present, and contains features of neuroinflammation, neuronal death, and skeletal muscle denervation [56]. Recently, complement activation has emerged as being closely related to the disease’s pathological progression. An early study revealed deposits of IgG and C3 immune complexes in the spinal cord and the motor cortex of ALS patients [41]. In a familial ALS rodent model using SOD1 G93A mice, intact C3 is present in both astrocytes and neurons, while cleaved C3b/iC3b exclusively localized in neurons at the symptomatic stages [19]. Meanwhile, increases in C1q and C3 are seen in the nerve–muscle junction of both transgenic ALS mice and post-mortem tissues of ALS donors [19,20]. However, C1q or C3 deletion does not change neuroinflammatory responses, ALS progression, or the survival in ALS mice [134].

Apart from C1q and C3, terminal complement proteins are beginning to attract research interests. Altered expression of complement components is observed in the spinal cord during the progression of ALS, including upregulation of C1qB, C4, factor B, C3/C3b, C5, and especially C5aR, and a decrease in CD55, CD59, with most of these changes occurring in the vicinity of microglia [77]. Similar to C1q and C3, C4 and MAC accumulate in the spinal cord and motor cortex of ALS human samples, along with reactive gliosis and peripheral immune cell infiltration [49]. Furthermore, the upregulation of C5a and MAC is identified in blood sampled from ALS patients [135]. Consistently, multiple complement components, including C5a and C5aR, accumulate in the spinal cord and skeletal muscles of different ALS rodent models, along with increased immune cell recruitment [58,59]. Either genetic or pharmacological inhibition of C5aR signaling dramatically decreases reactive glial cells, as well as the infiltration of macrophages and other immune cells in both the spinal cortex and skeletal muscle, which in turn attenuates neuronal death and improves muscle strength [56,57,59]. Thus, it appears that the C5a–C5aR signaling accelerates ALS progression via immune cell recruitment, but also provides another clue regarding the contributory role of MAC in ALS pathogenesis.

### 4.4. Parkinson’s Disease

PD is a progressive neurodegenerative condition characterized by loss of dopaminergic neurons in the basal ganglia with Lewy bodies. This disease mainly affects the motor system in its early stages and then gradually develops into non-motor symptoms such as dementia, depression, and sleep problems. The elevated transcription of C3 within reactive astrocytes is observed in the brain of PD patients [25]. Furthermore, another study reports that persistently high C3 and C4 levels in the serum from PD patients correlate with poorer neurological manifestations [136]. The absence of C1q or C3 exhibits little protective effect in PD animal models [42,43,85]. The CR3 knock-out mice are more resistant to neurodegeneration and motor dysfunction in the paraquat and maneb-induced PD model [64]. According to current evidence, whether complement proteins are involved in PD pathogenesis remains controversial.

### 4.5. Huntington’s Disease

HD is an inherited brain disorder caused by excessive trinucleotide repeats of CAG in the HTT gene. The symptoms consist of uncoordinated movements and cognitive problems with dementia-like symptoms. Compared with healthy individuals, the immunoreactivity of various complement proteins including C1q, C4, C3, and C9, is increased in neurons, myelin and astrocytes in brains affected by HD, with evidence of gliosis [21]. Elevated mRNA levels for these proteins are also detected using in situ hybridization, especially C3 and C9 from active microglia within the striatum [21]. Furthermore, pharmacological inhibition of C5aR signaling can attenuate weight loss, restore motor deficits, and reduce apoptotic cells within the lesions from an artificial, acute, and invasive HD rodent model [42,43]. Nevertheless, another more realistic transgenic mice model demonstrates that C3 deficiency does not protect against HD progression [44]. Thus, there is only a possible correlation of the presence of complement proteins with the development of HD.

### 4.6. Perioperative Neurocognitive Disorders

PNDs occur commonly after surgery and are characterized by a range of cognitive impairment, associated with poor recovery, reduced quality of life, and even high mortality. With emerging evidence showing increased levels of cytokines and glial cell activation in the postoperative brain, neuroinflammation has been implicated as a critical contributory factor to the development of PNDs [137,138]. Previous clinical studies have observed that various peripheral surgical trauma induces complement activation, including C3a, C5a, C5–C9, factor B, and other regulatory complement components in the blood [139,140,141,142]. Interestingly, C3 in astrocytes and C3aR in microglia are both increased in the brain after tibial fracture surgery, which further accelerates synapse loss, cognitive impairment, and blood–CSF barrier dysfunction [45]. Similarly, another study mimicking systemic inflammation by repeated intraperitoneal LPS injections demonstrates that cerebral complement activation is induced by systemic inflammation and is involved in dopaminergic neuron loss. These changes, however, were not seen in C3 deficient mice [143]. Thus, targeting C3 could be a promising therapeutic approach and warrants further investigation [138].

## 5. Therapies Targeting the Complement System

Targeting complement activation bears the therapeutic potential to minimize complement-mediated tissue damage that may occur in trauma, autoimmune diseases, neurological diseases, and neurodegenerative diseases. Currently, anti-complement agents are available which mainly inhibit convertase assembly and cleavage, MAC formation, and the C5–C5aR interaction. The clinical trials on neurological diseases mainly focus on the PNS, neuromuscular junction, and muscle. To date, agents targeting C5 provide the most successful therapeutic strategy, with complement treatment in myasthenia gravis (MG) and neuromyelitis optica spectrum disorders (NMOSD) being the most developed [85,144,145]. Here, we summarize current complement therapeutics and their clinical and preclinical evidence (Table 2).

### 5.1. Complement C1q, C1s, C1r Complex

ANX005, *a humanized anti C1q antibody*, shows a high affinity of binding activity with C1q to inhibit the classical complement pathway. While preclinical evidence reveals a protective effect in Guillain–Barré syndrome (GBS) and AD, its safety and tolerance are still under assessment [146].

*Sutimlimab*, also known as BIVV009, is a humanized antibody to C1s, which similarly prevents the activation of the classical pathway but preserves the C1q opsonic function. Several clinical trials were conducted on cold agglutinin disease (CAD) and paroxysmal nocturnal hemoglobinuria (PNH), but evidence remains lacking regarding neurodegenerative diseases [147,148,149,150].

### 5.2. Complement C3

*Intravenous immunoglobulin* (IVIg) is formulated from neutralizing autoantibodies. It also confers non-specific inhibition of the complement cascades, with its major effect considered to be against C3. IVIg has proven its beneficial effects of various diseases, such as MG, GBS, and MCI [144,145,151]. In addition, the experimental evidence shows a potential therapeutic value on stroke and AD [152,153,154,155].

*Compastatin* is a family of cyclic peptides, which binds to C3 to interfere with C3 convertase activity and C3 cleavage. APL-2 or percetacoplan is in different phases of clinical trials for PNH and age-related macular degeneration (AMD) [156,157]. A novel analog called AMY-101 has yet to reach clinical trials but is in early preparation [158].

### 5.3. Complement C5

*Eculizumab*, a humanized monoclonal antibody, has a high binding affinity to C5 and can block C5 cleavage and C5 convertase formation. It was approved by the FDA to treat PNH [144,156], and the effects on MG and NMOSD were tested in the phase 3 REGAIN and PREVENT clinical trials, respectively [160,161,162,163]. It was additionally tested in GBS, compared with the treatment of IVIg, in phase 2 [164].

*Ravulizumab* (ALXN1210) is another humanized monoclonal antibody, sharing a similar structure and function with *Eculizumab*, but has prolonged effects. It is also approved for PNH and has two ongoing phase 3 clinical trials for MG and NMOSD, respectively [165,166].

*Tesidolumab* and *SKY59* are both monoclonal antibodies, while SKY59 contains a longer half-life. Both of these agents neutralize C5 to prevent the activation of the terminal pathway and is undergoing phase 2 clinical trials for PNH [144,145].

*Zilucoplan* is an anti-C5 peptide that prevents C5b generation and MAC formation. The safety and efficacy have been evaluated in a phase 2 clinical study on MG [167].

*Cemdisiran* is an RNAi therapeutic targeting C5. The pharmacokinetic and pharmacodynamic properties were assessed on healthy volunteers and PNH patients [168].

### 5.4. Complement C5aR

PM53 and PM205 are both cyclic hexapeptides, acting as C5aR antagonists. Considering the protective effects observed from preclinical evidence, C5aR antagonists bear the potential to become clinical therapeutic agents [54,57,69,169,170].

## 6. Conclusions and Perspectives

The diverse role of the complement system in the CNS spans beyond immunity, and is only recently being increasingly appreciated. It is intertwined with the CNS from neurodevelopment to neurodegeneration. In the developing and healthy adult brain, tightly controlled regulation of the expression, function, and degradation of various complement proteins ensures appropriate complement activation, which maintains homeostasis and normal function of the brain. In contrast, dysregulation of the complement cascades promotes the onset of developmental and neurodegenerative diseases. In recent decades, the investigation of complement components in CNS pathologies has advanced dramatically. However, these studies mainly focus on the activator complement proteins and complement receptors rather than regulatory proteins, and the findings of different complement components in pathologies vary across different diseases. Therefore, a comprehensive picture of the complement system in molecular mechanisms of neurodevelopmental and neurodegenerative diseases still remains lacking.

Animal studies of neurogenerative diseases such as AD exhibit promising effects of complement inhibition or activation in protecting against cognitive decline, which bears potential as therapeutic targets. Nevertheless, these results are primarily limited to genetic manipulation of complement components and this review has summarized currently available anti-complement medications and their ongoing clinical trials, with the majority being engineered antibodies or peptides, with the only exception being RNAi in nature. The safety and efficacy of these agents were mainly evaluated in neurodegenerative diseases of the peripheral nervous system, neuromuscular junction or muscles. Experimental studies in CNS neurodegenerative diseases are lacking, let alone clinical trials. Given the physicochemical nature of currently available anti-complement agents, penetration into the CNS following peripheral administration may be problematic. Therefore, further research towards improving access across the BBB into CNS tissue is warranted.

Complement regulatory proteins maintain the balance of complement activation, which was tested in brain trauma to attenuate excessive inflammatory responses in order to minimize secondary cerebral damage and to improve cognitive function [4,171,172]. Thus, increasing the level of complement regulatory proteins to “neutralize” aberrant complement activation can provide a potential therapeutic option. Moreover, complement regulatory proteins expressed in neurons were shown to increase neuronal tolerance against complement attacks. Several novel endogenous complement inhibitors, which belong to the sushi domain family, were shown to protect neurons against excessive synapse elimination [106,107,173]. It may explain why susceptibility to complement attack varies between individuals.

In conclusion, the improved understanding of the intricacies of complement system in the CNS has opened a range of novel therapeutic possibilities against previously intractable diseases of the brain. Further work needs to be devoted to improving access of these agents to the CNS from peripheral administration to achieve the full therapeutic potential of these treatment approaches.

## Figures and Tables

**Table 1:** Complement expression in the CNS and their role in neurodevelopment and neurodegeneration.

Complement Component	Location	Role(s) in Normal Neurodevelopment	Pathophysiological Involvement in Neurodevelopmental and Neurodegenerative Diseases
C1q	neuron [9,10,11] microglia [2]	Synpatic pruning [9,12]	AD: mediates glial activation and promote synapse loss [13,14]
			ASD [15], MS [16,17,18], ALS [19,20], HD [21]
C3	astrocyte [22,23,24,25] microglia [2] neuron [10,11,26]	Progenitor proliferation [27], neuronal migration [28], Synaptic pruning [9]	ASD: mediates microglia synaptic pruning [29,30]
			AD: mediates microglial synaptic engulfment, direct neuronal toxicity, and Aβ clearance [31,32,33,34,35,36,37]
			MS: activates the alternative pathway, mediate microglia and synaptic engulfment [16,17,18,38,39,40]
			ALS [19,20,41], PD [42,43], HD [21,44]
			PNDs: related to increase microglial activation, neuronal loss, and BBB disruption [45]
C4	neuron [10,11,23,24]	-	Schizophrenia: each C4 allele increases the risk [46,47]
			ALS [48,49], HD [21]
C5	astrocyte [8,50] neuron [10,11,26]	Progenitor proliferation [51], neuronal migration [52]	AD: mediates pro-inflammatory responses [53,54,55]
			ALS: mediates pro-inflammatory responses [56,57,58,59]
			ASD [29]
MAC, C5-C9	astrocyte [50] neuron [10,11,26]	-	MS [60], ALS [49]
CR3	microglia [8,24]	Synaptic pruning [61,62]	AD: mediates microglial synaptic engulfment [32,37,63] PD: mediate microglial activation [64]
CR4	microglia [8,24]	-	-
C3aR	microglia [8,24] neuron [65,66] asotrycte [67]	Progenitor proliferation [27], neuronal migration [52]	AD:mediate microglial synaptic engulfment [35,68]
C5aR	microglia [8,24,69] neuron [65,70] astrocyte [71]	Progenitor proliferation [51], neuronal migration [52]	AD: mediates pro-inflammatory response [53,54]
			ALS: recruits immune cells including peripheral cell infiltration [56,57,58,59]
			MS: mediates pro-inflammatory response [60,72]
Crry	-	-	AD: anti-C3 inhibition and promotes Aβ plague formation [73]
			MS: anti-C3 inhibition and prevents synapse loss [38]
C1INH	astrocyte [24] neuron [2,24]	Neuronal migration [52]	MS [16]
MASP1, 2	-	Neuronal migration [28]	-
Factor H	astrocyte [24] microglia [74,75]	-	AD [76]
Factor B	astrocyte [22,24]	-	ALS [77]
Factor I	asotrycte [8]	-	-
C4BP	astrocyte [8]	-	-
CD55	astrocyte [8,66] neuron [2,24]	-	ALS [77]
CD59	asotrycte [8,66] neuron [2,24]	-	ALS [77]

MAC: membrane attack complex; AD: Alzheimer’s disease; ALS: amyotrophic lateral sclerosis; MS: multiple sclerosis; PD: Parkinson’s disease; HD: Huntington’s disease; PNDs: perioperative neurocognitive disorders.

**Table 2:** Complement Therapies, Current Clinical Development and Preclinical Studies on CNS Disease.

	Therapy	Drug Class	Mechanism	Approved Clinical Trials	Preclinical Study on CNS Disease
C1q	Anti-C1q antibody (ANX005)	Monoclonal antibody	Bind to C1q, inhibit Classical pathway	none	GBS, AD [146]
C1s	Sutimlimab (BIVV009)	Monoclonal antibody	Bind to C1s	CAD [147,148,149] PNH [150]	None
C3	high-dose IVIg	IgG	Unspecific, form complex with C3b, inhibit C3 convertase	Clinical trials on MG, GBS and others [144,145] MCI: [151]	Stroke: [152,153] AD: [154,155]
	Compstatin (APL-2 or Pegcetacoplan, AMY-101)	cyclic peptides	Bind to C3, interfere C3 convertase function and C3 cleavage	PNH: APL-2, Phase III, compared with eculizumab [156] AMD: phase 2 [157] Periodontitis: AMY-101, phase 2 [158]	none
C5	Eculizumab	Monoclonal antibody	Bind to C5, prevent C5 cleavage, inhibit MAC assembly	PNH: FDA-approved treatment; compared with Ravulizumab [159] MG: *REGAIN,* phase3 [160,161] NMOSD: *PREVENT,* phase3 [162,163] GBS: phase 2, compared with IVIg [164]	none
	Ravulizumab (ALXN1210)	Monoclonal antibody	Bind to C5, prevent C5 cleavage, inhibit MAC assembly	PNH: FDA-approved Treatment; compared with Eculizumab [159] MG: phase 3 [165] NMOSD: phase 3 [166]	none
	Tesidolumab	Monoclonal antibody	Neutralization of C5, Inhibit terminal complement activation	PNH: phase 2 [144]	none
	SKY59	Monoclonal antibody	Long-lasting Neutralization of C5	PNH: phase1/2 [145]	none
	Zilucoplan	peptide	prevents the cleavage of C5 into C5a and C5b	MG: phase 2 [167]	none
	Cemdisiran	RNAi	Suppress C5 production	PNH: pharmacological study [168]	none
C5aR	PMX53	cyclic hexapeptides	C5aR1 antagonists	none	I/R injury: [169]
	PMX205	cyclic hexapeptides	C5aR1 antagonists	none	AD: [54,69] ALS: [57,170]

CAD: cold agglutinin disease; PNH: Paroxysmal Nocturnal Hemoglobinuria; MG: myasthenia gravis; GBS: Guillain-Barré syndrome; MCI: mild cognitive impairment; AMD: Age-Related Macular Degeneration; NMOSD: neuromyelitis optica spectrum disorders; I/R injury: ischemia/reperfusion injury.

## Data Availability

Not applicable.
